# Azithromycin Susceptibility Testing and Molecular Investigation of *Neisseria gonorrhoeae* Isolates Collected in Russia, 2020–2021

**DOI:** 10.3390/antibiotics12010170

**Published:** 2023-01-13

**Authors:** Ilya Kandinov, Boris Shaskolskiy, Dmitry Kravtsov, Alexandra Vinokurova, Sofya Gorshkova, Alexey Kubanov, Victoria Solomka, Julia Shagabieva, Dmitry Deryabin, Ekaterina Dementieva, Dmitry Gryadunov

**Affiliations:** 1 Center for Precision Genome Editing and Genetic Technologies for Biomedicine, Engelhardt Institute of Molecular Biology, Russian Academy of Sciences, Moscow 119991, Russia; 2 State Research Center of Dermatovenerology and Cosmetology, Russian Ministry of Health, Moscow 107076, Russia

**Keywords:** *Neisseria gonorrhoeae*, azithromycin resistance, resistance determinants, efflux pump, *mtrR* and *mtrD* alleles

## Abstract

The aim of this work was to study the resistance to macrolides (azithromycin) in the modern Russian population of *N. gonorrhoeae* with the analysis of genetic resistance determinants. Azithromycin is not used to treat gonococcal infection in Russia. However, among 162 isolates collected in 2020–2021, 22 isolates (13.6%) were phenotypically resistant to azithromycin. Mutations in 23S rRNA genes were found only in two isolates; *erm* and *mefA* genes were absent. Azithromycin resistance was shown to be predominantly associated with mutations in the *mtrR* and *mtrD* genes of the MtrCDE efflux pump and their mosaic alleles which may have formed due to a horizontal transfer from *N. meningitidis*. A total of 30 types of *mtrR* alleles and 10 types of *mtrD* alleles were identified including mosaic variants. Matching between the *mtrR* and *mtrD* alleles was revealed to indicate the cooperative molecular evolution of these genes. A link between the *mtrR* and *mtrD* alleles and NG-MAST types was found only for NG-MAST 228 and 807, typical of *N. gonorrhoeae* in Russia. The high level of resistance to azithromycin in Russia may be related to the spread of multiple transferable resistance to antimicrobials regardless of their use in the treatment of gonococcal infection.

## 1. Introduction

Gonococcal infection is one of the most dangerous and widespread sexually transmitted infections (STIs). The causative agent of gonococcal infection is *N. gonorrhoeae*, a Gram-negative diplococcus, that is characterized by a high rate of both phenotypic and genetic adaptive changes. According to WHO data, about 82.4 million cases of gonorrhea were newly detected in 2020 [1]. Of particular concern is the ability of *N. gonorrhoeae* to rapidly accumulate mutations in the genome and acquire resistance to antimicrobial drugs [2,3].

Currently, most countries of the world use third-generation cephalosporins together with azithromycin as a dual antimicrobial therapy for the treatment of gonococcal infection [4,5,6]. Azithromycin is a macrolide class antibiotic used against a wide range of bacteria, including gonococcus. Macrolides cause the disruption of protein synthesis on the ribosomes of a microbial cell by binding to the 50S ribosomal subunit. The main binding sites are located at positions A2058, A2059, and C2611 in domains II and V of the 23S rRNA [7]. The expediency of the joint use of cephalosporins and azithromycin, which have different mechanisms of action, is explained by the increase in the efficacy of the therapy of gonococcal infection and by the eradication of another, most frequently detected STI pathogen, *Chlamydia trachomatis*, which is typically found as part of a mixed infection [6]. Gonococci with a reduced susceptibility to cephalosporins are found in the world population sporadically (0.1–2%), while the proportion of azithromycin-resistant *N. gonorrhoeae* isolates grows every year, and in some countries of Europe and Asia, it is close to 5%, which is the threshold value for the exclusion of this drug from treatment regimens in accordance with WHO recommendations [8,9].

The mechanisms of *N. gonorrhoeae* resistance to macrolides include specific chromosomal mutations, the presence of genes encoding rRNA methyltransferases, and the overexpression of efflux pumps [10]. Antibiotic-specific genetic determinants of resistance are mutations in the peptidyltransferase loop of the II and V domains of the 23S rRNA [11]. The C2611T substitution leads to the appearance of isolates with moderate resistance (minimum inhibitory concentration, MIC_Azm_ up to 4 mg/L), while the A2058G/A2059G substitution leads to highly resistant isolates (MIC_Azm_ > 256 mg/L) [12]. It should be especially noted that the level of resistance is also determined by the number of mutant 23S rRNA alleles. The *N. gonorrhoeae* genome contains four copies of the *rrn* operon (*rrnA*, *rrnB*, *rrnC*, *rrnD*), which include the nucleotide sequence encoding 23S rRNA. In particular, isolates with three or all four mutant alleles have been shown to be highly resistant (MIC_Azm_ ≥ 256 mg/L and up to 4096 mg/L), while isolates with only one copy of the mutant 23S rRNA allele have an insignificant level of resistance (MIC_Azm_ = 0.25–1 mg/L) [13], i.e., below or at the threshold separating resistant and susceptible forms (ECOFF MIC_Azm_ ≤ 1 mg/L) according to the criterion of the 2022 European Committee on Antimicrobial Susceptibility Testing (EUCAST) [14].

Other mechanisms of *N. gonorrhoeae* resistance are associated with efflux systems that ensure the outflow of antimicrobial drugs, including macrolides, from the cell. The MtrCDE efflux system is encoded by the *mtrC-mtrD-mtrE* operon. Its activation is associated either with changes in the functioning of the MtrCDE transcriptional repressor, the *mtrR* gene [15,16], or with changes in the configuration of MtrCDE proteins [17]. For the *mtrR* gene, the Ala39Thr, Arg44His, Gly45Asp, and Ala86Thr substitutions, and the *delA* deletion at position −35 in the promoter region and/or insertion of the *insT/instTT* nucleotides at position −10 of *mtrR* leads to an increase in the expression of the MtrCDE efflux pump and the removal of antimicrobial drugs, including macrolides, out of the cell. Isolates with such mutations have a reduced phenotypic susceptibility to azithromycin. Mosaic alleles of the MtrCDE efflux pump operon appear as a result of a horizontal transfer between closely related *Neisseria* species, such as *N. meningitidis*, *N. sicca*, *N. lactamica*, *N. cinerea*, *N. flavescence*, etc. Mosaic variants of the *mtrR* and *mtrD* genes are capable of activating the MtrCDE efflux system and, respectively, the removal of azithromycin from the cell, which leads to an increase in the MIC_Azm_ to 8 mg/L or more [17]. Mosaic variants of the promoter region of the *mtrR* gene (Meningitidis-like (MG-like) promoter) have also been described; as a result of mosaicity, the repression of the entire MtrCDE operon may be impaired, which leads to an increase in the MIC_Azm_ up to 8 mg/L [18,19].

The outflow of macrolides from the bacterial cell and a decrease in susceptibility to macrolides is also associated with the gonococcus efflux pump encoded by the *mefA* conjugative gene. It has been shown that the *mef* gene, which was originally found in Gram-positive microorganisms, can be transferred and expressed in Gram-negative microorganisms, including *N. gonorrhoeae* [20,21].

Another factor affecting the susceptibility of *N. gonorrhoeae* to macrolides is the synthesis of 23S rRNA methyltransferases ErmA, ErmB, ErmC, and ErmF. These enzymes cause methylation of the adenine A2058 in the peptidyltransferase loop of the V domain of the 23S rRNA, that leads to the modification of the antibiotic target, a decrease in binding affinity, and the formation of azithromycin resistance (MIC_Azm_ ≥ 8–64 mg/L) [11]. The presence of *erm* genes is associated with an increase in the level of phenotypic susceptibility of *N. gonorrhoeae* to macrolides; however, at present, 23S rRNA methylases are rarely found in resistant isolates [22], for example, the PubMLST database includes only three isolates with *erm* genes.

In the Russian Federation, azithromycin has never been used for the treatment of gonococcal infections because the proportion of resistant isolates has consistently exceeded the WHO recommended threshold of 5% since the starting of large-scale screening for susceptibility to this antibiotic in 2007 [23]. At the same time, the reasons for the decrease in the susceptibility of *N. gonorrhoeae* to azithromycin in the Russian population when this antibiotic is not used in gonorrhea treatment regimens are unclear, and the mechanisms that determine azithromycin resistance have not been studied in detail.

The goal of this work was to study the resistance of *N. gonorrhoeae* to macrolides (azithromycin) in the modern Russian population of the pathogen (2020–2021) with the analysis of the genetic determinants of resistance and the reasons for their spread.

## 2. Results

### 2.1. Phenotypic Azithromycin Susceptibility and Analysis of Genetic Determinants Associated with Azithromycin Resistance

A total of 162 *N. gonorrhoeae* clinical isolates collected in different regions of the Russian Federation in 2020–2021 were analyzed; MIC_Azm_ and genetic determinants associated with azithromycin resistance were determined. Among these isolates, 22 (13.6%) were found to be azithromycin-resistant (MIC_Azm_ > 1 mg/L, up to 8 mg/L) and 140 (86.4%) isolates were azithromycin-susceptible (MIC_Azm_ = 0.01–1.0 mg/L).

Only two isolates carried mutations in the 23S rRNA gene. MIC_Azm_ of both isolates was 4 mg/L, which was due to C2611T mutations in all four copies of the 23S rRNA gene. In 160 isolates (98.7%), mutations in the MtrCDE efflux pump genes were found, leading to amino acid substitutions both in the *mtrR* gene and its promoter region, and in the *mtrD* gene. In some cases, an increase in the expression of the MtrCDE efflux pump and, as a consequence, the formation of resistance (MIC_Azm_ ≈ 2 mg/L) were due to the mosaic structure of the *mtrR* and/or *mtrD* genes. No isolates containing the *erm* genes encoding Erm methyltransferases, and the *mefA* efflux pump gene were found. Characteristics of the studied isolates including *mtrR* and *mtrD* allele types, NG-MAST type, MIC_Azm_, mutations in the *mtrR*, *mtrD*, and 23S rRNA genes, are provided in Supplementary Materials, Table S1.

Thus, the resistance to azithromycin in the analyzed sample of *N. gonorrhoeae* isolates was only sporadically associated with mutations in the 23S rRNA gene and was mainly formed as a result of an increase in the expression of the MtrCDE efflux pump, and this was the rationale for carrying out in-depth analyses of the *mtrR* and *mtrD* genes.

### 2.2. Diversity of the mtrR Gene Alleles

**Figure 1:**
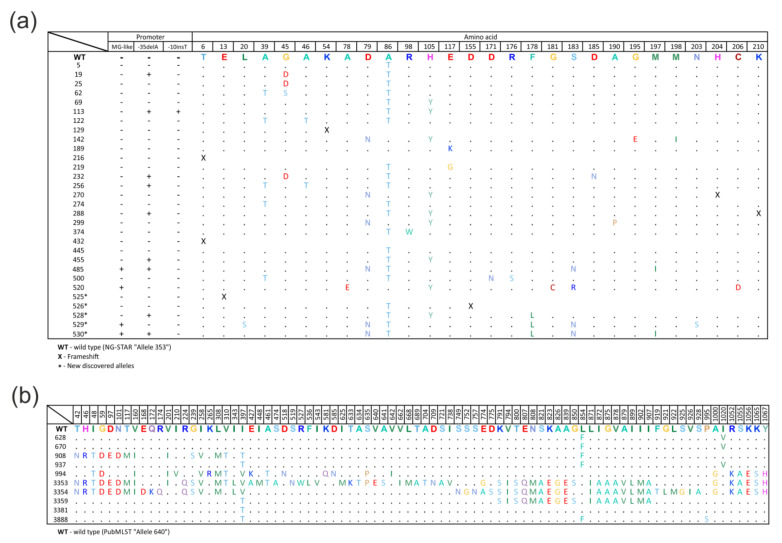
Amino acid sequences of *mtrR* (**a**) and *mtrD* (**b**) alleles in *N. gonorrhoeae* isolates collected in the Russian Federation in 2020–2021 in comparison with the wild type (WT) *mtrR* (NG-STAR “Allele 353”) and *mtrD* (PubMLST “Allele 640”) sequences. Designations: ‘+’ – presence of deletion/ MG-like promoter; ‘–‘ – absence of deletion/ MG-like promoter.

The types of *mtrR* alleles were determined for the first time in the Russian population of the causative agent of gonorrhea. A total of 30 different types of *mtrR* alleles were identified in 162 clinical isolates (Figure 1a). Among them, there were no wild type (NG-STAR “Allele 353”) alleles.

Most of the alleles had 2–4 amino acid substitutions compared with the wild type sequence. The most common substitutions were Ala39Thr (29.0%), Gly45Asp (6.2%), Gly45Ser (7.4%), Ala86Thr (89.5%), and His105Tyr (17.9%), either separately or in various combinations. In total, 43 isolates with mutations in the promoter region of the *mtrR* gene were identified; all of these isolates had an adenine deletion at position −35 from the start codon (-35delA), and one isolate additionally had a thymine insertion at position −10 (-10insT).

Of particular note is that 26 isolates (16%) had alleles associated with the mosaic structure of the *mtrR* gene which were probably formed as a result of horizontal transfer from *N. meningitidis* (NG-STAR Alleles “485”; “436”; “520”). A mosaic structure in these alleles was found both in the coding sequence of the *mtrR* gene (from four to six amino acid substitutions) and in its promoter region (MG-like promoter) [18].

Five novel *mtrR* alleles that were not present in the databases were identified. All new sequences were submitted to the NG-STAR database and assigned the following numbers:

- allele 525—insertion of nucleotide A at position 38, resulting in a frameshift;
- allele 526—deletion of two GA nucleotides at position 465 of the gene, resulting in a frameshift;
- allele 528—T→G substitution at position 531, which does not lead to amino acid substitution;
- allele 529—T→C substitution at position 132, resulting in the Cys44Arg amino acid substitution;
- allele 530—T→G substitution at position 531, which does not lead to amino acid substitution, and T→C substitution at position 535, resulting in the Phe178Leu amino acid substitution.

### 2.3. Diversity of the mtrD Gene Alleles

A total of ten variants of *mtrD* alleles were identified (Figure 1b); among them, there were no wild type alleles (NEIS1633 “Allele 640”). Some types of alleles differed only in the nucleotide sequence, i.e., they had synonymous substitutions that did not lead to a change in the amino acid sequence.

The most common were alleles 908 (26%), 628 (24%), and 937 (23%), which had 2–15 amino acid substitutions in comparison with the wild type sequence. However, we also found alleles of the *mtrD* gene that arose as a result of a probable horizontal transfer from *N. meningitidis* (NEIS1633 Alleles “3353”; “3354”; “3359”). These mosaic allele types had 19–62 amino acid substitutions and were found in 36 isolates, comprising about 20% of the entire sample. Of particular note is that all mosaic types were found to have Ser821Ala and Lys823Glu substitutions which were most often associated with a decrease in susceptibility to macrolides [17].

### 2.4. Relationship between the mtrR and mtrD Alleles

**Figure 2:**
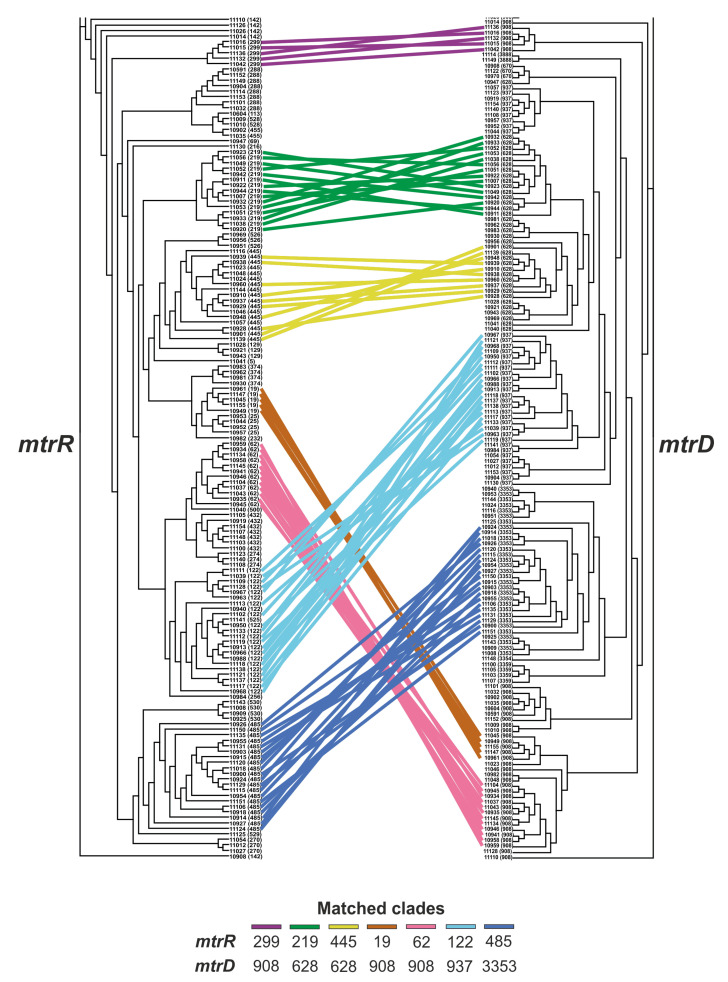
Correspondence between *mtrR* and *mtrD* cladograms constructed independently for the Russian *N. gonorrhoeae* isolates collected in 2020–2021. Sample numbers and the *mtrR* or *mtrD* allele numbers are indicated on the branches. Matched clades are formed by linked leaves of the phylogenetic tree. Matched clades consisting of five or more isolates are painted in different colors.

Our analysis revealed matching between different alleles of the *mtrR* and *mtrD* genes. For example, twenty *N. gonorrhoeae* isolates carrying *mtrR* allele 122 also carried *mtrD* allele 937; in twelve isolates, *mtrR* allele 62 was accompanied by *mtrD* allele 908 (Figure 2 and Supplementary Table S2). The integration of the results of phylogenetic analysis for the *mtrR* and *mtrD* genes with data on the matching of alleles allowed us to identify the matching clades. In total, 25 matched clades with linked *mtrR* and *mtrD* alleles were found in the isolate sample; out of them, 7 clades included 5–20 isolates (shown on Figure 2) and 18 clades included 2–4 isolates (not shown on Figure 2 but presented in Supplementary Table S2). Thus, for the first time for the Russian population of *N. gonorrhoeae*, we demonstrated the co-evolution of the *mtrR* and *mtrD* genes.

Of particular note is the clade of isolates with linked *mtrR* 485 and *mtrD* 3353 alleles. It includes twenty isolates in which nucleotide sequences from *N. meningitidis* were found both in the *mtrR* gene and its promoter, and in the *mtrD* gene. This cluster was one of the most numerous in terms of the number of isolates. Due to the presence of mosaic structures in the *mtrR* and *mtrD* genes, isolates from this cluster proved to be resistant to azithromycin (MIC_Azm_ ≈ 2 mg/L).

The second largest clade consisted of fifteen isolates with linked *mtrR* 219 and *mtrD* 628 alleles. It is formed exclusively by isolates with NG-MAST sequence types 807 and 228, which are the most common in the Russian population of *N. gonorrhoeae* [24]. This clade of isolates, although one of the most numerous, has not been associated with azithromycin resistance.

### 2.5. Effect of Genetic Profile on MIC_Azm_

To systematize the obtained data, 162 studied *N. gonorrhoeae* isolates were divided into seven groups according to the median MIC_Azm_ values and mutations in the *mtrR* and *mtrD* alleles, and *rrn* operon (Table 1):

- group I: 12 isolates with the single His105Tyr substitution in the *mtrR* gene;
- group II: 47 isolates, 98% of which had the Ala86Thr mutation in the *mtrR* gene;
- group III: 35 isolates with His105Tyr and Ala39Thr substitutions in the *mtrR* gene. Several isolates carried the Gly45Ser mutation;
- group IV: 23 isolates with the His105Tyr substitution (100%) and the -35delA mutation in the promoter region of the *mtrR* gene (83%). There were also the -10insT, Ala39Thr, and Gly45Asp mutations;
- group V: 13 isolates with the His105Tyr (100%) and Ala39Thr (54%) substitutions in the *mtrR* gene and the Ser821Ala, Lys823Glu substitutions in the *mtrD* gene associated with mosaic structure (92%);
- group VI: 23 isolates with nucleotide sequences derived from *N. meningitides* both in the *mtrR* gene and its promoter (MG-like promoter), and in the *mtrD* gene (Ser821Ala and Lys823Glu mutations). There was also the -35delA mutation in the promoter region of the mtrR gene and the Ala86Thr mutation in the *mtrR* gene;
- group VII: two isolates with the C2611T mutations in all four alleles of the 23S rRNA gene. One isolate contained an MG-like promoter, the -35delA, Ala39Thr, Ala86Thr mutations in the *mtrR* gene, and the Ser821Ala, Lys823Glu mutations in the *mtrD* gene;
- ungrouped (7 isolates).

The median value of MIC_Azm_ for isolates from group I was 0.06 mg/L, and the single His105Tyr mutation detected in these isolates, apparently, makes a minimal contribution to the formation of resistance. Isolates from groups II–IV demonstrated only a slight decrease in susceptibility to azithromycin (MIC_Azm_ = 0.12 mg/L).

The median value of MIC_Azm_ for isolates from group V was 0.25 mg/L. It should be noted, however, that this group includes azithromycin-resistant isolates (five out of thirteen) with MIC_Azm_ of 2–8 mg/L. The decreased susceptibility to azithromycin within this group is most likely associated with the Ser821Ala and Lys823Glu mutations in the *mtrD* gene.

The formation of resistance to azithromycin within group VI with the median value of MIC_Azm_ of 2 mg/L is probably associated with the MG-like promoter, the simultaneous presence of -35delA and Ala86Thr mutations in the *mtrR* gene, and the Ser821Ala and Lys823Glu mutations in the *mtrD* gene associated with its mosaic structure.

**Figure 3:**
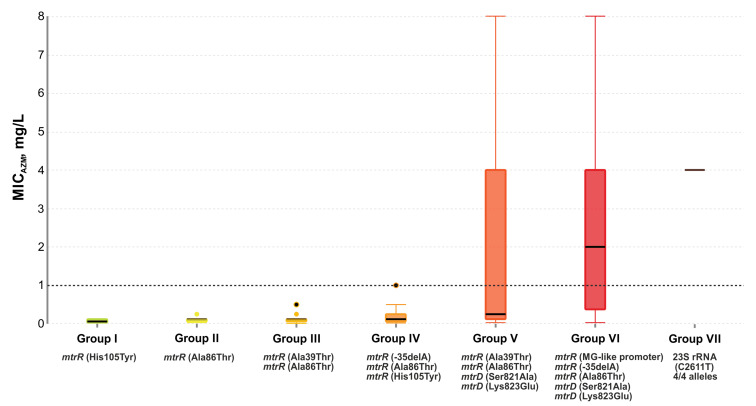
Box plot: MIC_Azm_ in groups of *N. gonorrhoeae* isolates with different genetic profiles. A description of the profiles by group is given in Table 1. Thick solid lines in each box indicate the median MIC_Azm_ value for each group. The whiskers extend to the boundaries of the interquartile range. The dotted line indicates the EUCAST breakpoint for azithromycin resistance.

The highest median value of MIC_Azm_ = 4 mg/L was observed in group VII and was associated with the C2611T mutations in all four alleles of the 23S rRNA gene. The box plot diagram of changes in MIC_Azm_ depending on the genetic profile in groups is shown in Figure 3.

### 2.6. Phylogenetic Tree for mtrR and mtrD Genes

**Figure 4:**
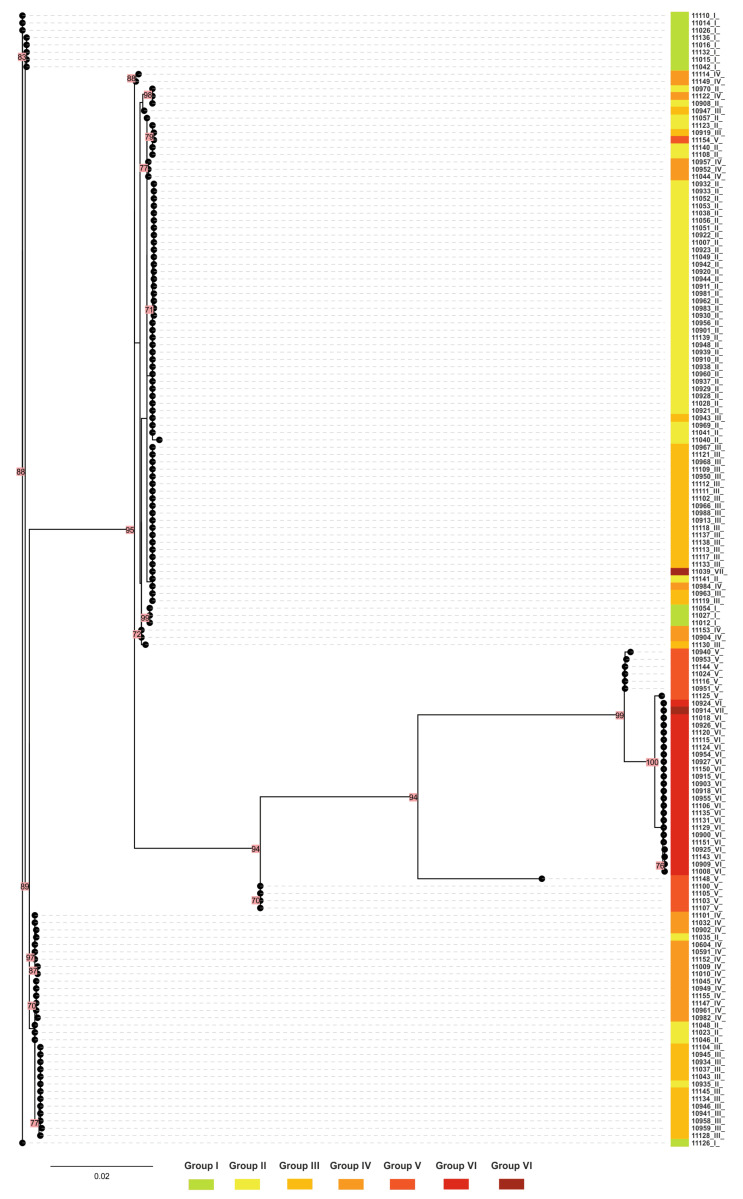
Maximum likelihood phylogenetic distance tree for concatenated *mtrR* and *mtrD* gene sequences of the *N. gonorrhoeae* isolates collected in Russia in 2020–2021. Isolate numbers are colored according to groups I-VII (Table 1, Figure 3). Bootstrap values of 70 or more are colored in red.

For alleles of the *mtrR* and *mtrD* genes found in the Russian *N. gonorrhoeae* population, a maximum likelihood phylogenetic tree (phylogram) was constructed (Figure 4). The group colors correspond to those in the box-plot diagram (Figure 3). Isolates on the phylogram form clades in accordance with the division into groups I-VII described above. This tree demonstrates the relative phylogenetic proximity of isolates from groups I-IV, in which no azithromycin-resistant isolates were found. Isolates from groups V and VI are located phylogenetically far away from groups I-IV and form a separate cluster in the tree. This remoteness is due to a large number of substitutions in the *mtrR* and *mtrD* genes associated with their mosaic structure, which also affects the resistance to azithromycin within these groups (MIC_Azm_ ≈ 2 mg/L) (Table 1, Figure 4).

### 2.7. MtrR and mtrD Alleles in Isolates Belonging to NG-MAST 228 and 807

The NG-MAST types were determined for all the studied isolates (Table S1). In general, we did not see any relationship between molecular types and *mtrR* and *mtrD* gene types for our sample of isolates. The exceptions were isolates belonging to MG-MAST 228 and 807, i.e., those types of sequences that, as has been previously established [24], were most common in the Russian Federation. These sequence types belong to the G807 genogroup, and the combined *porB* and *tbpB* sequences of NG-MAST 228 and 807 differ by one nucleotide. The isolates were characterized by susceptibility to azithromycin and belonged to group II (Table 1). Almost all the isolates of these types had the same allele of the *mtrD* gene, allele 628, and similar alleles of the *mtrR* gene. To test the assumption about the relationship between the types of *mtrR* and *mtrD* alleles and NG-MAST types, we expanded the sample of isolates by adding the isolates of NG-MAST 228 and 807 collected in Russia in 2016–2019, for which the whole genome sequencing was performed by us previously (GenBank, Bioproject PRJNA768989). In total, the sample included 41 Russian isolates of *N. gonorrhoeae* collected in 2016–2021 (Table 2).

Almost all Russian isolates of NG-MAST 228 and 807 (40 out of 41 isolates) had the *mtrD* allele 628, and 39 out of 41 isolates carried *mtrR* alleles 5, 445, 526, and 537, differing from allele 219 by one nucleotide (Table 2). Thus, indeed, a link was revealed between the NG-MAST 228 and 807, typical of the Russian *N. gonorrhoeae* population, and the types of *mtrR* and *mtrD* alleles.

## 3. Discussion

Despite the fact that azithromycin is not used for the treatment of gonococcal infection in Russia, the resistance of *N. gonorrhoeae* to this antibiotic is at a high level. According to the EUCAST criteria, only 86.4% of the clinical isolates collected in the Russian Federation in 2020–2021 were phenotypically susceptible to this antimicrobial, which is below the WHO recommended threshold of 95% for its use. This situation is especially paradoxical since the proportion of resistant isolates in neighboring countries remains negligible. In Russia’s neighbor Belarus, where azithromycin is also not used to treat gonorrhea, the proportion of azithromycin-resistant isolates in 2018–2019 was only 2.6% [25]. At the same time, in Kyrgyzstan, for which the similarity of *N. gonorrhoeae* populations with those in Russia was noted (the presence of a large number of isolates of NG-MAST 1751 and 807), no isolates resistant to azithromycin were found in 2017, although azithromycin, along with ceftriaxone, is recommended for the treatment of gonococcal infections [26].

To clarify the nature of *N. gonorrhoeae* resistance to azithromycin in Russia, we analyzed the genetic determinants of resistance. We identified polymorphisms in the 23S rRNA, *mtrR*, and *mtrD* genes, including their mosaic variants, while the *erm* and *mefA* genes were found to be missing in the genomes of the isolates. Antibiotic-specific mutations in all four copies of the 23 rRNA genes were found only in two isolates (1.2% of the analyzed sample). Thus, resistance to azithromycin in the studied sample of isolates was mainly due to nucleotide substitutions in the genes encoding the MtrCDE efflux pump (non-specific mechanism of resistance), namely, mutations in the *mtrR* and *mtrD* genes, and their mosaic alleles, which were formed in the course of a probable horizontal gene transfer from *N. meningitidis*. The obtained results allowed us to conclude that the mechanism of azithromycin resistance associated with the MtrCDE efflux pump arose in the Russian population of *N. gonorrhoeae* as a means of counteracting other antimicrobial drugs (e.g., penicillin, tetracycline). Thus, the increased level in resistance to azithromycin in the Russian population of *N. gonorrhoeae* may be associated with the wide spreading of non-specific mechanisms of resistance to antimicrobial drugs that have emerged in the population regardless of the use/non-use of this antibiotic.

An important observation is the matching between the *mtrR* and *mtrD* alleles, as a result of which, 25 matching clades with linked alleles were found in the Russian population of *N. gonorrhoeae*. Of particular note are matching clades which simultaneously contain mosaic variants of both *mtrR* and *mtrD* genes. Thus, we revealed the cooperative molecular evolution of the *mtrR* and *mtrD* genes in the Russian population of *N. gonorrhoeae*. The demonstrated linkage shows that recombination events between closely related *Neisseria* spp. can, apparently, affect both individual genes and entire operons.

Previously, we have shown a functional dependence between the *penA* allele type and the NG-MAST, i.e., isolates of the same sequence type carried the same *penA* alleles, although the opposite was not true [27]. In this study, a link between the *mtrR* and *mtrD* alleles of the efflux pump and NG-MAST was found only in isolates of the sequence types 228 and 807 that are typical of the Russian population of *N. gonorrhoeae*. It can be noted that these isolates did not have the determinants of azithromycin resistance and they mainly carried the *mtrR* alleles 445/219 and the *mtrD* allele 628. In this work, we did not perform detailed phylogenetic study of *N. gonorrhoeae* isolates and comparative population analysis with European isolates in order to identify similar clades of azithromycin-resistant isolates, and this will be the subject of a separate study.

The reason for the emerging azithromycin resistance in the Russian gonococcal population and the formation of a separate cluster of resistant isolates can most likely be due to several reasons: (1) the cross-border transfer of a resistant strain with specific resistance determinants from abroad, where such strains are already found everywhere [16] and resistance in such strains has been developed during the treatment of gonococcal infection with azithromycin; (2) a recombination event between *N. gonorrhoeae* and closely related *N. meningitidis* followed by its fixation in the population [28,29] as a mechanism of resistance non-specific to azithromycin.

## 4. Materials and Methods

### 4.1. Collection and Characterization of N. gonorrhoeae Isolates

For the characterization of the modern Russian population of *N. gonorrhoeae*, clinical isolates of *N. gonorrhoeae* (162 samples) were collected in 2020–2021 from 7 subjects of the Russian Federation (Arkhangelsk, Astrakhan, Kaluga, Omsk Oblasts, City of Moscow, Stavropol Krai, Chuvash Republic) by the State Research Center for Dermatovenerology and Cosmetology of the Ministry of Health of Russia. The samples were provided by specialized medical organizations of the dermatovenerological profile, each sample from an individual patient. Sample collection, transportation, culturing, phenotyping, and storage were performed according to the protocols described previously [23,30].

### 4.2. Testing of N. gonorrhoeae Susceptibility for Azithromycin

Measurement of the minimum inhibitory concentration for azithromycin (MIC_Azm_) was performed by the serial dilutions in chocolate agar. Each strain was characterized in accordance with the EUCAST (ECOFF) criteria for azithromycin: S—susceptible (MIC_Azm_) < 1.0 mg/L), R—resistant (MIC_Azm_) > 1.0 mg/L).

### 4.3. Study of Genetic Determinants of N. gonorrhoeae Resistance to Azithromycin

The sequences of all four copies of the 23S rRNA gene in *N. gonorrhoeae* samples were determined by Sanger sequencing as described previously [11]. The *mtrR* gene sequences were determined by Sanger sequencing using the NG-STAR protocol (https://ngstar.canada.ca/, accessed on 26 September 2022) with the previously described primers [31].

The *mtrD* gene was amplified using PCR with For1-5′-ACGGCATCTGAAGCCAAA-3′ and Rev1-5′-AAAGTCCTGATGCCGTCTG-3′ primers. The resulting 3304 bp amplicons were Sanger sequenced using additional primers For2-5′-CGGCAACGTCATCCTCCG-3′ and Rev2-5′-TGTAACCGCCGCCCAATT-3′. The obtained nucleotide sequences, two from the forward primer and two from the reverse primer, were concatenated using the Unipro UGENE v.44 program [32].

The study of *N. gonorrhoeae* isolates for the presence of the *ermA/B/C/F* methyltransferase genes and the *mefA* gene, was performed as described previously [20].

### 4.4. Determination of mtrR and mtrD Allele Types

The resulting nucleotide sequences for each gene were combined into a multiple alignment file in the Bio-Edit program (Ibis Biosciences, Carlsbad, CA, USA) and checked for mutations, stop codons, or frameshift. The wild type sequence PuBMLST “Allele 640” was used as a reference genome for *mtrD* allele alignment. As noted in [33], there is no consensus about which amino acid, Tyr or His, is present in codon 105 of the wild type *mtrR* sequence. In this study, we took the sequence NG-STAR “Allele 353”, GenBank accession number KT954121.1, as a wild type for *mtrR*, i.e., this sequence containing His at codon 105.

For each isolate in the sample (162 isolates), the *mtrR* and *mtrD* allele numbers were determined. For the *mtrR* alleles, the NG-STAR database (https://ngstar.canada.ca, accessed on 26 September 2022) was used; for *mtrD* alleles, the BIGSdb database (https://pubmlst.org, accessed on 10 October 2022) was used according to the “NEIS1633(*mtrD*)” typing scheme. If there was not a complete match between the analyzed sequence and the gene sequence in the database, for example, the sequence under study contained a mutation, a stop codon, or a frameshift, we submitted that sequence into the database as a new one.

### 4.5. NG-MAST Typing

Molecular typing of *N. gonorrhoeae* isolates was performed using the standard NG-MAST protocol [34]. The variable internal regions of the *porB* and *tbpB* genes were PCR-amplified and the resulting products were purified and sequenced using a 3730xl Genetic Analyzer (Applied Biosystems, Waltham, MA, USA). Both the leading and reverse strands were assessed and the sequencing data were processed using the 3730/3730xl Data Collection Software version 3.0. Allele numbers for the *porB* and *tbpB* sequences and sequence types were assigned according to the NG MAST v2.0 database (https://pubmlst.org, accessed on 20 October 2022).

### 4.6. Construction of Phylogenetic Trees

Phylogenetic trees were constructed using the RaxML v.8.2.4 software (https://usegalaxy.eu/, accessed on 17 October 2022) with 1000 rapid bootstrap inferences. Cladograms for the *mtrR* and *mtrD* genes were constructed independently from each other according to the sequences of alleles corresponding to each isolate. To construct a phylogram, we used concatenated *mtr* and *mtrD* gene sequences obtained using the Bio-Edit alignment tool (Ibis biosciences, Carlsbad, CA, USA).

## Figures and Tables

**Table 1:** Genetic profiles of *N. gonorrhoeae* isolates.

Group (Number of Isolates)	Median MIC_Azm_ (mg/L)	Genetic Profile *mtrR*									Genetic Profile *mtrD*			23S rRNA
		*mtr* Allele	MG-Like Promoter	-35delA	-10insT	Ala39 Thr	Gly45 Asp	Gly45 Ser	Ala86 Thr	His105 Tyr	*mtrD* Allele	Ser821 Ala	Lys823 Glu	C2611T (4 Alleles)
I (12)	0.06	299 (42%) 142 (33%) 270 (25%)	–	–	–	–	–	–	–	100% *	908 (75%) 937 (25%)	–	–	–
II (47)	0.12	219 (33%) 445 (30%) 374 (9%) 274 (6%) 129 (4%) 142 (4%) 526 (4%) 5 (2%) 62 (2%) 455 (2%) 500 (2%) 525 (2%)	–	–	–	11%	–	2%	**98%**	–	628 (74%) 908 (11%) 937 (11%) 670 (4%)	–	–	–
III (35)	0.12	122 (57%) 62 (31%) 129 (3%) 69 (3%) 432 (3%) 216 (3%)	–	–	–	**94%**	–	31%	**100%**	6%	937 (60%) 908 (34%) 628 (6%)	–	–	–
IV (23)	0.12	288 (36%) 19 (22%) 25 (13%) 528 (9%) 113 (4%) 142 (4%) 232 (4%) 256 (4%) 455 (4%)	–	**83%**	4%	4%	39%	–	**100%**	**52%**	908 (61%) 937 (26%) 3888 (9%) 670 (4%)	–	–	–
V (13)	0.25	432 (45%) 445 (23%) 25 (8%) 122 (8%) 526 (8%) 529 (8%)	8%	–	–	**54%**	8%	–	**100%**	–	3353 (54%) 3359 (30%) 937 (8%) 3354(8%)	**92%**	**92%**	–
VI (23)	2.0	485 (83%) 530 (17%)	**100%**	**100%**	–	–	–	–	**100%**	–	3353 (100%)	**100%**	**100%**	–
VII (2)	4.0	485 (50%) 122 (50%)	50%	50%	–	50%	–	–	**100%**	–	3353 (50%) 937 (50%)	50%	50%	**100%**
Ungrouped (7)	1	129 (30%) 142 (14%) 299 (14%) 219 (14%) 189 (14%) 520 (14%)	–	–	–	–	–	–	43%	43%	908 (33%) 628 (33%) 994 (17%) 3381 (17%)	–	–	–

* Cells, where the percentage of occurrence of a particular mutation in the group exceeded 50%, are highlighted in bold.

**Table 2:** Characteristics of *N. gonorrhoeae* isolates belonging to the sequence types most common in the Russian Federation.

NG-MAST	Number of Isolates	*mtrR* Allele	*mtrD* Allele	Comment
228	4	219	628	Alleles *mtrR* 5, 445, 526, 537 differ from allele *mtrR* 219 by one nucleotide
	4	445	628	
	2	5	628	
	3	537	628	
	1	62	908	
807	14	219	628	
	3	445	628	
	3	526	628	
	6	5	628	
	1	500	628	

## Data Availability

sequences of the new *mtrR* alleles were added to the NG-STAR database under accession numbers: allele 525, 526, 528, 529, 530.

## Supplementary Materials

The following supporting information can be downloaded at: https://www.mdpi.com/article/10.3390/antibiotics12010170/s1, Table S1: Characteristics of *N. gonorrhoeae* isolates collected in the Russian Federation in 2020–2021; Table S2: Link between *mtrR* and *mtrD* allele types of *N. gonorrhoeae* isolates collected in the Russian Federation in 2020–2021.
